# Antimicrobial-resistant Bacteria Arising from the Use of Colistin Sulfate in the Livestock (2nd edition) (Antimicrobial-resistant Bacteria)

**DOI:** 10.14252/foodsafetyfscj.D-21-00003

**Published:** 2021-03-30

**Authors:** Food Safety Commission of Japan

## Abstract

The Food Safety Commission of Japan (FSCJ) updated a risk assessment on antimicrobial-resistant bacteria arising from the use of a veterinary medicinal product, colistin sulfate, in cattle and pigs, according to the “Assessment Guideline for the Effect of Food on Human Health Regarding Antimicrobial-Resistant Bacteria Selected by Antimicrobial Use in Food-producing Animals” (FSCJ, September 30, 2004). Both *Escherichia coli *(*E. coli*) and *Salmonella enterica *subsp*. enterica *(*Salmonella*) were potential antimicrobial-resistant bacteria. In cases of occurrences of human infectious diseases due to the bacteria in foods derived from livestock, these resistant bacteria could be responsible for reduction or loss of the antibiotic treatment efficacy. FSCJ thus conducted a risk assessment of *E. coli* and *Salmonella* as identified hazards. FSCJ judged to be low on the occurrence probability and extent of selection of drug-resistant *E. coli* and *Salmonella*, due to the use of colistin sulfate in cattle and pigs, unless otherwise the use of colistin increases. The chance and extent of human exposure to the resistant bacteria were evaluated low via livestock products including pigs and cattle, as long as proper cooking practice is implemented. The degree of possible reduction or loss of clinical effectiveness against *E. coli* and *Salmonella* was evaluated as moderate. The overall estimation of the risk regarding reduction or loss of clinical effectiveness of antimicrobials in humans was low. It is necessary to keep up with the latest scientific findings and information.

## Conclusion in Brief

The Food Safety Commission of Japan (FSCJ) updated a risk assessment on antimicrobial-resistant bacteria arising from the use of a veterinary medicinal product, colistin sulfate, in cattle and pigs, according to the “Assessment Guideline for the Effect of Food on Human Health Regarding Antimicrobial-Resistant Bacteria Selected by Antimicrobial Use in Food-producing Animals” (FSCJ, September 30, 2004).

In the first assessment (2017), *Escherichia coli* (*E. coli*) was identified as a hazard^1^^)^. Based on the assessment, the Ministry of Agriculture, Forestry and Fisheries (MAFF) withdrew the designation of colistin as a feed additive, and placed restriction on its use for veterinary therapeutics in 2018.

In addition to *E. coli*. in the 1st edition, *Salmonella enterica* subsp. *enterica* (*Salmonella*) is newly added as a hazard in the 2nd edition. In fact, monitoring on *E. coli* and *Salmonella* derived from livestock, conducted from 2000 through 2017, has suggested the retention of colistin susceptibility in almost all these bacteria. Some strains were found to contain *mcr*-1, *mcr*-3, and/or *mcr*-5 genes in these samples.

### Hazard Identification

Colistin sulfate is an antibiotic polypeptide that has been used in cattle, pigs and poultry in Japan since 1950s. Colistin’s use had been suspended clinically in humans due to the frequent adverse effects such as renal dysfunctions, and its role has been covered newly developed substitute antibiotics. In 2015, a formula of colistin methanesulfonate for injections was re-approved owing to the situation where infections with multidrug-resistant gram-negative bacilli became clinical issues in recent years.

Mechanisms of two-component regulatory systems associated with chromosomal genes have been known to operate for colistin-resistance in gram-negative bacteria. In 2015, *E. coli* strains harboring a gene (*mcr*-1) associated with colistin-resistance on plasmids were reported in China. Up to now, colistin-resistant gene families of *mcr*-1 through 10 have been known within colistin-resistant families. The susceptibilities of *E. coli* and *Salmonella* against colistin remain high in Japan, based on the monitoring results of these bacteria isolated from the livestock from 2000 through 2017. Some strains carrying the *mcr*-1, *mcr*-3 and/or *mcr*-5 genes were, however, found among the isolates^2^^)^.

Both *E. coli* and *Salmonella* were potential antimicrobial-resistant bacteria to be selected under the use of colistin sulfate in cattle and pigs. In cases of occurrences of human infectious diseases due to the bacteria in foods derived from livestock, these resistant bacteria could be responsible for reduction or loss of the antibiotic treatment efficacy. FSCJ thus conducted a risk assessment of *E. coli* and *Salmonella* as identified hazards.

### Release Assessment

FSCJ judged the probability of selection of drug-resistant *E. coli* and *Salmonella* is low, due to the use of colistin sulfate in cattle and pigs. The following points were discussed prior to obtaining the conclusion; 1) The prevalence of *mcr*-gene was 2.0% and below in *E. coli* derived from domestic healthy livestock and in *Salmonella* derived from the sick livestock in 2015, although *mcr*-gene would be transferred among *E. coli* and between other bacteria belonging to enterobacteriaceae^2^^)^, 2) fitness cost of bacteria with acquisition of plasmid harboring *mcr*-gene was confirmed, 3) the use of colistin was currently prohibited as a feed additive, and colistin was recognized as a second-line drug in 2018, and 4) finally, the increased rates of colistin-resistance among *E. coli* and *Salmonella* derived from livestock will be low unless otherwise the use of colistin increases.

### Exposure Assessment

*E. coli* and *Salmonella* are occasionally detected in meats. However, colistin-resistant strains were scarcely isolated from these products. Considering proper cooking of the livestock products derived from cattle and pigs, the chance and extent of human exposure to the resistant bacteria were evaluated low via livestock products.

### Consequence Assessment

The influence on clinical effectiveness was evaluated as moderate. Clinical importance of colistin is high as it is a recommended drug against multidrug-resistant gram-negative bacillus infections including carbapenem-resistant *Enterobacteriaceae* (CRE) infection caused by *E. coli*. Reports of multidrug-resistant gram-negative bacillus infections such as those by CRE in human cases are limited at present in Japan. Carbapenem-resistant strain has not been found among *E. coli* derived from domestic livestock. Human exposure to both colistin- and carbapenem-resistant *E. coli* from domestic animals through foods is thus unlikely at present. While *E. coli* or *Salmonella* may transfer *mcr*-gene to other bacteria, multidrug-resistant (MDR) gram-negative bacillus such as CRE acquire colistin-resistance through such *mcr*-gene transfer. As a result, other effective antibiotics may be limited. Especially, in the case of MDR *Pseudomonas aeruginosa* (MDRP), impact on the treatment will be significant, because colistin is often used to treat MDRP infectious disease in humans. However, transfer of *mcr*-gene to MDRP was not determined in conjugal-transfer test using *mcr*-gene harboring *E. coli* as a donor. Therefore, the possibility that MDRP acquires colistin-resistance by transfer of *mcr* gene from livestock derived colistin resistant *E.coli *or *Salmonella* is low at present. 

Antibiotics are not used generally for *Salmonella*-induced enteritis. The use of colistin is also not recommended for severe cases of systemic *Salmonella* infection under the use of other antibiotics.

### Risk Characterization

Considering the hazard identification, the release assessment, the exposure assessment, and the consequence assessment, the overall estimation of the risk was low. This assessment was conducted on the premise of risk management measures taken by the MAFF in 2018. It is necessary to keep up with the latest scientific findings and information.
